# The combination of psychosocial working conditions, occupational balance and sociodemographic characteristics and their associations with no or negligible stress symptoms among Swedish occupational therapists – a cross-sectional study

**DOI:** 10.1186/s12913-021-06465-6

**Published:** 2021-05-18

**Authors:** Carita Håkansson, Annika Lexén

**Affiliations:** 1 Division of Occupational and Environmental Medicine, Lund University, Lund, Sweden; 2 Department of Health Sciences, Lund University, Lund, Sweden

**Keywords:** Burnout, Health profession, Psychosocial factors, Work-life balance

## Abstract

**Background:**

The numbers of people who are on sick leave due to mental health problems, such as exhaustion disorder, are increasing in Sweden. One of the most affected groups is healthcare professionals. In order to develop preventive strategies to promote a sustainable working life for occupational therapists, there is a need to understand the associations between psychosocial working conditions, occupational balance and no or negligible stress symptoms. To the best of our knowledge, neither the combination of these factors nor the salutogenic perspective, have been taken into consideration when exploring factors associated with stress symptoms among occupational therapists in previous research.

**Methods:**

Purposeful sampling was used. All currently working members of the Swedish Association of Occupational Therapists were invited to take part in the present study in 2018. The final sample was 3658 (48% response rate). A web-survey with questions about signs of exhaustion, psychosocial working conditions, occupational balance and sociodemographic characteristics was used. Logistic regression analyses were used in order to calculate associations between psychosocial working conditions, occupational balance, sociodemographic characteristics and no or negligible signs of exhaustion.

**Results:**

Experiencing high occupational balance, low workload, high control, high sense of community and high justice, were associated with no or negligible stress symptoms. Furthermore, a satisfying financial situation, having children living at home, being married and long professional experience were significant in this model.

**Conclusions:**

It seems important to consider not only psychosocial working conditions, but also occupational balance to prevent exhaustion disorder among occupational therapists in Sweden. Organisational programmes, which reduce the workload, strengthen the control and sense of community and facilitate occupational balance, seem to be important areas for health promotion in this group.

## Introduction

The numbers of people who are on sick leave due to mental health problems, such as exhaustion disorder, are increasing in Sweden [1]. Exhaustion disorder is thought to develop from chronic stress [2]. One of the most affected groups is healthcare professionals [3]. In 2008, paramedical service providers, including occupational therapists, had the highest proportion of exhaustion of all healthcare professionals [4]. A newly performed descriptive study [5] of the same Swedish occupational therapists studied in the present study, showed that they rated their workload as high, their reward as low. Furthermore, they pointed out a value incongruence between their and the workplaces’ values and did not experience a good occupational balance. One-fifth of them met the diagnostic criterion for mild or pronounced exhaustion disorder. In order to develop preventive and intervention strategies to promote a sustainable working life for occupational therapists, there is a need to understand the associations between psychosocial working conditions, occupational balance and no or negligible stress symptoms.

Studies of occupational therapists have shown that a high workload was associated with having an exhaustion disorder [6, 7]. Additionally, in a study of Swedish occupational therapists [8] “working at a superficial level due to lack of time” was associated with a high level of perceived stress. Poulsen et al. [7] also found an association between low psychological detachment from work during out of work hours and exhaustion disorder among occupational therapists. Bringing work home after work and not switching off mentally during off-job time can mean that it is impossible to replenish resources that are essential for work-related recovery, such as spending time in activities that replenish resources. This can be interpreted as a lack of occupational balance.

Occupational balance is defined as the individual’s subjective experience of having the right amount of, and the right variation between different activities in daily life, i.e. work, home and family activities, leisure, rest and sleep [9]. Occupational imbalance has been shown to be a contributing factor for developing mental health problems, such as an exhaustion disorder among healthcare workers [10]. Additionally, a study of midwives showed that midwives who reported a lack of satisfaction with their occupational balance were more likely to report an exhaustion disorder [11].

Previous research also indicates that sociodemographic factors may be a contributing factor for developing mental health problems. In a study by Poulsen et al. [7], occupational therapists with 6–10 years of professional experience, aged 25–35 years, or not having children, had a high risk of an exhaustion disorder. However, previous research of occupational therapists also showed that those with children had a higher risk of exhaustion disorder than those without children [12]. Associations between physical activity and perceived stress also have been shown among people in the general population [13] and between financial hardship factors and perceived stress among parents [14].

In summary, it seems that it is of importance to consider not only psychosocial working conditions, but also occupational balance, family situation, years of professional experience, financial situation and physical activity when exploring factors associated with no or negligible stress symptoms among occupational therapists. To the best of our knowledge, neither the combination of these factors nor the salutogenic perspective, have been taken into consideration, when exploring factors associated with stress symptoms among occupational therapists in previous research.

The aim of the present study was to explore associations between the combination psychosocial working conditions, occupational balance sociodemographic characteristics and no or negligible stress symptoms among Swedish occupational therapists.

## Method

The present study is a cross-sectional study, data was collected with a web-survey and analysed using logistic regression.

### Participants

Purposeful sampling was used. All currently working members of the Swedish Association of Occupational Therapists were invited to take part in the present study conducted in 2018 (*n* = 7600). Being on sick leave or parental leave, or being unemployed were exclusion criteria. All of them received an information letter and 3658 gave their written informed consent to take part giving a response rate of 48%. This sample is representative of Swedish occupational therapists concerning age and gender (Personal communication Martin Östberg, the Swedish Occupational therapists association). The characteristics of the participants are described in Table 1 and for more details see Lexén, Kåhlin, Erlandsson and Håkansson [5].

**Table 1:** Characteristics of the participants (*n* = 3658)

	n (%)
*Gender*	
Women	3332 (95)
Men	186 (5)
*Married/living with someone*	
Yes	2836 (80)
No	691 (20)
*Children living at home*	
Yes	1942 (56)
No	1543 (44)
*Physical activity*	
Mainly sedentary	442 (13)
Physically active	2933 (87)
*Financial situation*	
Satisfied	1695 (50)
Not satisfied	1703 (50)
*Signs of exhaustion (LUCIE)*	
No or negligible lasting stress symptoms	1953 (60)
Mild lasting stress symptoms	712 (22)
Moderate lasting stress symptoms	357 (11)
Severe lasting stress symptoms	214 (7)
*Occupational balance (OBQ11)*	
Median	13
Min-max	0–33
Q1-Q3	9–19

### Data collection

A web-survey was used. A reminder to complete the survey was sent on two occasions [15], 3 and 6 days after the first survey. In the present study, questions about signs of exhaustion (Lund University Checklist for Incipient Exhaustion), psychosocial working conditions (QPS-mismatch), occupational balance (Occupational Balance Questionnaire) and sociodemographic characteristics were used.

*Lund University Checklist for Incipient Exhaustion* (LUCIE) assesses signs of exhaustion and not fully developed exhaustion disorder [16]. LUCIE consists of 28 items in 6 domains; 1) Sleep and recovery, 2) Separation between work and leisure, 3) Sense of community and social support at the workplace, 4) Managing work duties and personal ability, 5) Private life and leisure activities and 6) Health complaints. Each domain consists of questions about “For the past month, to what extent have you felt or observed the following?” A four-point response scale is used, ranging from not at all (1) to very much (4). LUCIE consists of two scales: the Stress Warning Scale (SWS) and the Exhaustion Warning Scale (EWS). SWS reflects milder signs of exhaustion and EWS reflects more severe signs of exhaustion. The scores on each scale range from 0 to 100. A SWS score ≤ 17.00 (the SWS green zone) indicates no or negligible lasting stress symptoms. A SWS score between 17.01 and 38.50 (the SWS yellow zone) suggests possible mild lasting stress symptoms. A SWS score ≥ 38.51(the SWS red zone) indicates moderate lasting stress symptoms. An EWS score ≤ 21.50 (the EWS green zone) indicates that signs of exhaustion are absent or negligible. A high EWS score > 21.50 (the EWS red zone) suggests severe signs of exhaustion that might indicate exhaustion disorder. These two scales are combined into four steps showing the severity of the stress;

- Step 1-SWS green zone and EWS green zone = no or negligible lasting stress symptoms
- Step 2-SWS yellow zone and EWS green zone = possible mild lasting stress symptoms
- Step 3-SWS red zone and EWS green zone = moderate lasting stress symptoms, but less severe than exhaustion disorder
- Step 4-SWS red zone and EWS red zone = lasting stress symptoms of a severity indicating possible exhaustion disorder [17].

In the present study the LUCIE scores were dichotomized into: 1 = no or negligible stress symptoms (i.e., LUCIE step 1), and 0 = mild, moderate and severe stress symptoms indicating exhaustion disorder (i.e., LUCIE steps 2–4).

LUCIE has shown good content validity [17], good convergent validity [16] and good test-retest reliability [18].

*Psychosocial working conditions* were assessed using QPS-mismatch, which is a shorter form of QPS Nordic [19, 20]. The development of the shorter QPS mismatch was intended to provide an approximate picture of an individual’s mismatch between psychosocial working conditions and his/her ability. The questions in QPS-mismatch have been grouped according to Maslach and Leiter’s model of burnout development [21] into the following groups or dimensions: 1) Workload, 2) Control, 3) Community, 4) Reward, 5) Justice and 6) Values. QPS-mismatch has five response alternatives ranging from very seldom or never (1) to very often or always (5). The reliability was shown to be good (Cronbach alpha 0.72–0.90 in all dimensions) [Österberg, 2016, personal communication]. For each dimension the z-values were used. The z-values are based on the differences from the QPS Nordic reference group average score [19]. In the present study the z-values were dichotomised into low (0) versus high (1).

*Occupational balance* was measured with the Occupational Balance Questionnaire (OBQ11) [22]. The OBQ11 consists of 11 statements such as “I balance the different kinds of activities in my life, e.g., work, household chores, leisure, rest, and sleep” and “There is a balance between activities that give me energy versus those that drain my energy”. The statements have four response categories, ranging from not agreeing at all (0) to fully agreeing (3). The total sum score (0–33) shows the level of occupational balance, from low to high. In the present study, the QBQ11 sum score was divided into quartiles in the analysis. The OBQ has been proven to have good content validity, good internal consistency, and sufficient test–retest reliability [23] and good construct validity [22].

*Sociodemographic factors* included were years of professional experience, married/living with someone (yes/no), children living at home (yes/no), physical activity, and financial situation (satisfied/not satisfied). Physical activity was measured with one question “How physically active have you been the last three months”, dichotomised into mainly sedentary versus physically active (light/moderate or heavy).

### Statistical analysis

The multicollinearity between the independent variables was evaluated by Spearman’s rank correlation and the correlation coefficients were not too great (< 0.7) [24].

First, univariate logistic regression analyses were done between psychosocial working conditions, occupational balance, sociodemographic factors and no or negligible stress symptoms. In the next step, the independent factors with a *p*-value < 0.1 were selected for a multivariate analysis.

Odds Ratios (OR) and 95% Confidence Intervals (CI) were calculated, and the level of significance was set to *p* < .05. All the statistical analyses were performed using the SPSS program, version 26.

## Results

### Associations between psychosocial working conditions, occupational balance and no or negligible stress symptoms

Bivariate associations were found between all variables, and no or negligible stress symptoms (Table 2).

**Table 2:** Bivariate associations between psychosocial working conditions, occupational balance, sociodemographic factors and no or negligible stress symptoms

	OR (95% CI)
Low workload	5.73 (4.89–6.72)
High control	5.37 (4.59–6.28)
High reward	2.63 (2.25–3.07)
High community	4.01 (3.45–4.67)
High justice	3.81 (3.27–4.43)
Value congruence	3.24 (2.78–3.77)
High occupational balance	3.25 (2.47–3.55)
Long professional experience	1.30 (1.13–1.50)
Being married	1.34 (1.12–1.59)
Had children living at home	1.07 (0.93–1.23)
Physical active	2.28 (1.86–2.80)
Satisfying financial situation	2.31 (2.00–2.67)

The multivariate associations between psychosocial working conditions, occupational balance, sociodemographic factors and no or negligible stress symptoms, are shown in Table 3. Experiencing high occupational balance, low workload, high control, high sense of community and high justice were associated with no or negligible stress symptoms. Additionally, being married, having children living at home, satisfying financial situation and long experience were significant in this model.

**Table 3:** Multivariate associations between psychosocial working conditions, occupational balance sociodemographic factors and no or negligible stress symptoms

	OR (95% CI)
Low workload	**2.62 (2.11–3.27)**
High control	**1.76 (1.39–2.23)**
High reward	1.07 (0.84–1.35)
High community	**1.68 (1.32–2.15)**
High justice	**1.54 (1.18–2.00)**
Value congruence	1.19 (0.94–1.50)
Occupational balance^a^	
Q1	**3.72 (2.80–4.93)**
Q2	**8.59 (6.27–11.79)**
Q3	**19.09 (13.12–27.80)**
Long professional experience	**1.33 (1.08–1.65)**
Being married	**1.33 (1.02–1.73)**
Had children living at home	**1.35 (1.09–1.65)**
Physical active	1.34 (0.99–1.83)
Satisfying financial situation	**1.56 (1.27–1.93)**

^a^Occupational balance was divided into quartiles

## Discussion

This is the first nationwide survey conducted to determine the combination of psychosocial working conditions, occupational balance and sociodemographic characteristics associations with no or negligible stress symptoms among Swedish occupational therapists. The results showed that high occupational balance and psychosocial working conditions, such as low workload, high sense of community, high control and high justice were associated with no or negligible symptoms of stress.

In the present study, the strongest association was found between high occupational balance and no or negligible stress symptoms. A possible explanation to this result could be that occupational therapists are mainly women, and previous studies among women have shown associations between occupational balance and good subjective health [25–27]. Swedish women still take the main responsibility for home and family [28]. The double burden of employment and home and family activities, means that the balance between these activities and recovery is extra important in order to prevent stress [29].

Associations were also found between psychosocial working conditions and stress symptoms. As expected, based on previous studies, a strong association was found between workload and stress symptoms among occupational therapists [6–8] and mental health personnel [30], between control and stress symptoms among district nurses [31] and between justice perceptions and stress symptoms among nurses [32]. Another important working condition factor in the present study, was a high sense of community. Community, in the present study, is about relationships at the workplace and about being able to receive support and help from colleagues and supervisors, as well as about stress and conflict in these relationships [19]. Community buffers against the negative effects of to high work demands [33] and has been shown to be associated with health, job satisfaction and productivity [34]. Furthermore, the association in the present study between a high sense of community and no or negligible stress symptoms confirms the results of previous studies among other health professionals, i.e. psychiatrists [35] and nurses [36]. This can be interpreted as good relationships at the workplace are important to employee wellbeing.

However, to the best of our knowledge, only one previous study has been done focusing on psychosocial working conditions, occupational balance, sociodemographic factors and stress symptoms among psychiatrists in Japan [37]. But this study only investigated work life balance and two aspects of the psychosocial working environment, i.e. work-environment satisfaction and social support at the workplace, and their associations with exhaustion. To some extent the results of the present study confirm these authors’ results, but the results of the present study also add more aspects of the psychosocial working environment as well as physical activity, professional experience and financial situation.

### Methodological considerations

One limitation of the present study was its cross-sectional design, as it cannot reveal causality. Further studies with a longitudinal design are therefore needed.

Another limitation is the rather low response rate (48%), which could lead to selection bias [38]. However, concerning age and gender this sample is representative of Swedish occupational therapists (Personal communication Martin Östberg, the Swedish Association of Occupational Therapists, www.sverigesarbetsterapeuter.se). Furthermore, this response rate is similar to the response rate in other large-scale surveys [39]. Further, there is no reason to believe that the sample is not representative in terms of the associations investigated.

In the present study, variables related to psychosocial working conditions, occupational balance and sociodemographic were included. There are, of course, other stressors that can affect the degree of stress, such as if the workplace has been reorganised but also low occupational status. This possible shortcoming in the present study suggests the need for more research, including these variables.

A further limitation is that QPS mismatch has not been sufficiently tested for validity and reliability. We were aware of this, but still considered this to be a good instrument with which to measure working conditions. QPS-Nordic, from which the instrument was developed, has been found to have sufficient psychometric properties among employees in Sweden [40]. However, the lack of psychometric evidence for QPS-Mismatch needs to be taken into consideration when interpreting the results.

Furthermore, the reader should keep in mind the choice not to adjust for multiple testing. A significance level of 5% was used, meaning that 1 in 20 tests would be statistically significant by chance. In total, we tested around 13 hypotheses in the present study, and the risk of type I error is low. Therefore, multiple testing was not a major reason for interpreting the results with caution [41].

## Conclusions

It is important to consider not only psychosocial working conditions, but also occupational balance to prevent lasting stress symptoms indicating a possible exhaustion disorder among occupational therapists in Sweden. To reduce the workload, strengthen the sense of community and facilitate occupational balance seem to be important areas for health promotion in this group.

## Data Availability

The dataset used and analysed during the current study is available from the corresponding author on reasonable request.

## Abbreviations

LUCIE: Lund University Checklist for Incipient Exhaustion
EWS: Exhaustion Warning Scale
SWS: Stress Warning Scale
OBQ11: Occupational Balance Questionnaire with 11 items
